# A Novel Rolling Driving Principle-Enabled Linear Actuator for Bidirectional Smooth Motion

**DOI:** 10.34133/cbsystems.0424

**Published:** 2026-01-09

**Authors:** Dunfa Long, Fujun Wang, Chengzhi Hu, Chaoyang Shi

**Affiliations:** ^1^ Key Laboratory of Mechanism Theory and Equipment Design of Ministry of Education, School of Mechanical Engineering, Tianjin University, Tianjin 300072, China.; ^2^ Department of Mechanical and Energy Engineering, Southern University of Science and Technology, Shenzhen 518055, China.

## Abstract

This work presents a novel rolling driving principle (RDP) for stick-slip actuators to achieve high motion consistency, inspired by the rack-and-pinion mechanism. This RDP utilizes a symmetrical driving structure and tangential contact to realize the pure rolling motion between the stator and the slider, requiring just a single lead zirconate titanate (PZT). This configuration ensures a consistent bidirectional driving process with a constant contact force, which improves both motion consistency and linearity. Based on this RDP principle, a linear stick-slip actuator incorporating an isosceles trapezoidal flexible mechanism (ITFM) has been implemented. The corresponding driving principle, operating principle, and the RDP’s advantages have been analyzed and revealed. Design optimization was performed to investigate the optimal structural parameters of the ITFM. The superior performance of the proposed RDP-type actuator was experimentally verified across both high- and low-frequency ranges. The results indicate that the presented design exhibits forward and reverse output speed values of 0.410 and 0.417 mm/s at 10 Hz with linear correlation coefficients of 0.99969 and 0.99962, indicating an excellent motion consistency with a velocity difference ratio of 1.96%. When working at 560 Hz, the presented actuator reaches 37.73 and 34.99 mm/s for the forward and reverse output speed, yielding high linearity values of 0.99999 and 0.99999 due to the tiny speed fluctuation, and maintains a reasonable motion consistency with a velocity difference ratio of 7.54%. Finally, an RDP-type actuator-based magnetic resonance imaging (MRI)-compatible microsurgical instrument was proposed and prototyped, which enables opening–closing motions and cutting motions for intraoperative MRI surgical applications.

## Introduction

Piezoelectric actuators have played an essential role in precision positioning, micro- and nano-scaled fabrication, optical assembly and focusing, and micro-nano robotic systems owing to their merits of high precision, fast response speed, large force generation, and compact design [1–6]. However, the limited travel range, typically around ^1^/_1,000_ of their length, commonly restricts their usage [6–10]. To overcome this, various stepping-based actuation mechanisms have emerged, including inchworm actuators [11,12], ultrasonic actuators [13,14], and stick-slip actuators [15–19], each offering the potential for infinite strokes. The inchworm actuator requires coordinating multiple independent piezoelectric elements, resulting in complex structures and control strategies. The ultrasonic actuator typically operates at resonant frequencies and suffers from severe wear and heat issues. In contrast, the stick-slip actuator offers the advantages of simple structure and control and demonstrates great promise for practical applications [17–19].

Several stick-slip actuators have been proposed based on the impact-inertial driving principle [20,21], the stick-slip motion principle [22,23], and the parasitic motion principle (PMP) [16,24–27]. Among them, the PMP-type actuators demonstrate excellent output speed and load capacity and thus receive a surge of attention [25–27]. However, their asymmetric structure can complicate achieving consistent motion in both directions, which affects the actuator’s precision and repeatability, and makes bidirectional control more difficult [28]. The discontinuities in their stepping motion pose a challenge for achieving high linearity in movement [28,29].

Currently, there is a lack of research focused on improving bidirectional motion consistency. The majority of existing studies employed symmetric PZT-driven structures for high bidirectional motion consistency [30–32]. Wang et al. [30] achieved improved bidirectional consistency by symmetrically arranging 4 sets of compliant driving units in 2 pairs, which generated forward and reverse motions, respectively. The experimental results showed a displacement deviation of about 18.5% (120 V, 1 Hz). However, the multiple driving units increase the actuator’s size and require complex and cumbersome manual pre-adjustments to achieve a highly symmetrical assembly, due to the machining and assembly errors. To simplify the assembly and manual pre-adjustment processes, Tang et al. [31] proposed an X-shaped flexible mechanism-based actuator that used a single driving foot for bidirectional motion, reducing the adjustment difficulties compared with the multiple driving feet. Experimental results showed an optimal displacement deviation of 1.6% (100 V, 10 Hz). Huang et al. [32] devised an actuator with a symmetric structure and an optimized installation method, and this actuator employed rotating bearings for assembly to improve adaptive adjustment and streamline the adjustment process. The optimal displacement deviation was tested to be 6.8% (100 V, 10 Hz). Both of these 2 studies utilize 2 PZTs, resulting in a complex system with a large size, while the adjustment method needs further simplification for the bidirectional motion consistency. Furthermore, the bending-type actuator utilized symmetrical structures driven by ceramic plates [33,34]. Xu et al. [34] proposed a symmetrical flexible hinge mechanism and a stick-slip actuator driven by 2 ceramic plates, demonstrating an excellent bidirectional consistency for forward and reverse motions. However, the ceramic plates limit the actuator’s maximum speed (2,195.29 μm/s) and its maximum load capacity (1.1 N). In summary, while PZT-driven stick-slip actuators typically exhibit better overall output performance [32], further investigation is required to achieve high bidirectional consistency without additional adjustment and minimize the actuator’s complexity by minimizing the number of PZTs.

To enhance output motion linearity and achieve a smooth motion, one effective approach is to minimize or suppress backward motion. This approach has been extensively researched but has limited improvement in motion linearity. Tang et al. [35] managed to reduce the contact force between the stator and slider during the slip stage by adding an extra PZT-driven structure for the actuator’s adjustment. A sequential control method was applied for operations, which resulted in a minimal or even zero backward motion ratio. However, its motion linearity remains poor, and the extra PZT leads to a larger and more complicated structure and requires a complex control method. Yang et al. [36] designed an arc-shaped flexure hinge to form a PMP-type actuator and utilized the forward friction from the hinge’s elastic recovery to counterbalance the reverse friction in the slip stage. The backward motion was eliminated when the initial gap between the stator and slider was greater than 130 μm. However, the motion linearity needs further improvement, and the initial gap reduces the self-locking capability and stability of the actuator. Deng et al. [29] constructed a 2 degree-of-freedom (DOF) motion platform with four 2-DOF PZTs by guiding the driving foot to follow a circular path. This design allowed for bidirectional motion with only a stick stage in a circular operation. Experiments demonstrated that the backward motion was eliminated, but the low linearity remained because the motion was intermittent. Therefore, the motion linearity needs further investigation to be improved.

**Fig. 1.**
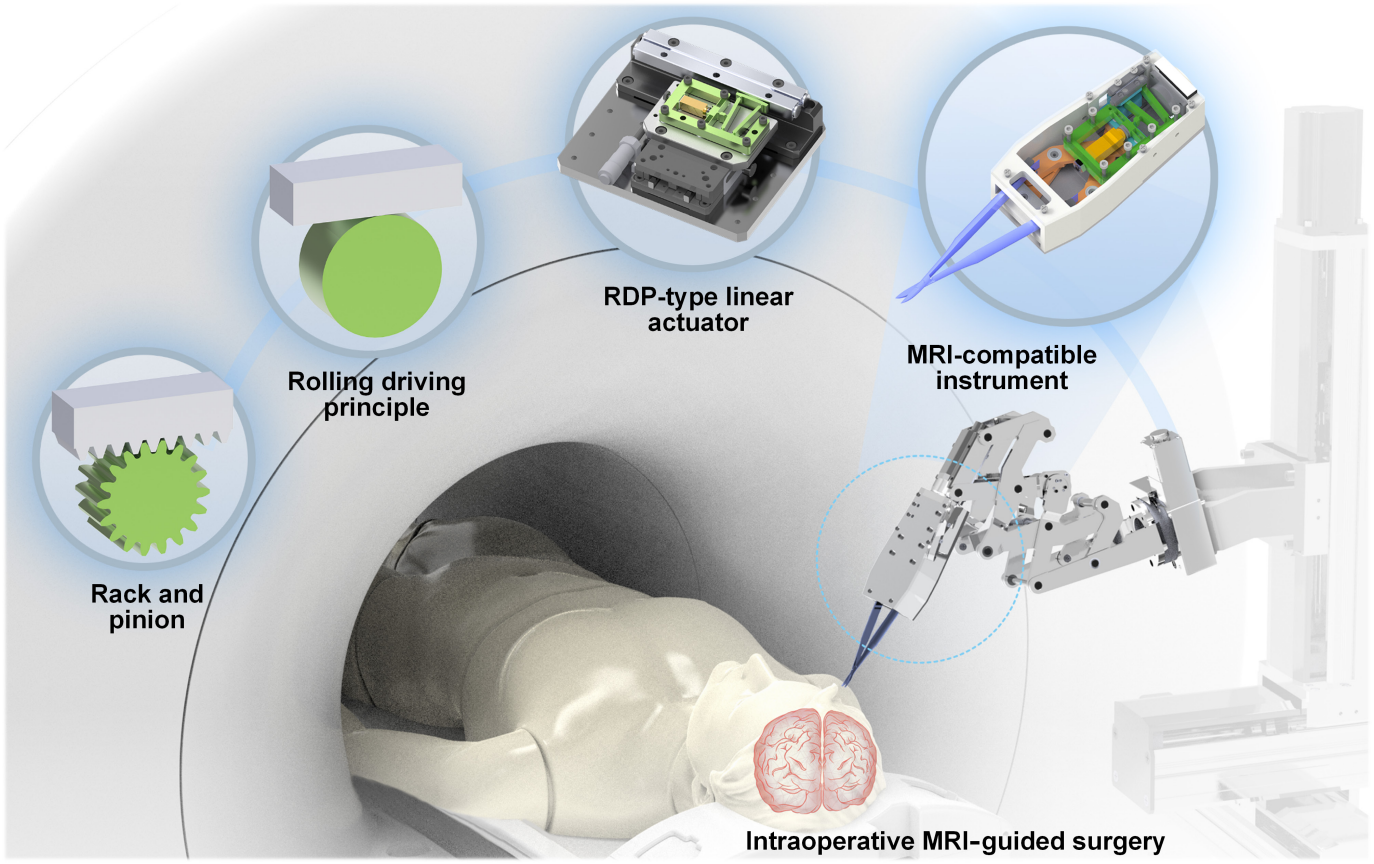
The proposed RDP-type stick-slip actuator and its application in MRI-compatible instruments with great potential for intraoperative MRI-guided surgery.

To achieve high bidirectional motion consistency and linearity, a novel rolling driving principle (RDP) inspired by the rack-and-pinion mechanism has been proposed. The RDP’s symmetrical driving structure and tangential contact ensure consistent bidirectional driving motion and require no manual pre-adjustment processes because of its self-adaptation to assembly errors. Meanwhile, the constant contact force between the stator and slider enables the reduction of speed fluctuations and enhances the higher motion linearity. Based on this RDP, an innovative linear stick-slip actuator with an isosceles trapezoidal flexible mechanism (ITFM) has been designed. Experiments demonstrated that the prototyped actuator achieved bidirectional smooth motion with excellent consistency and linearity driven using just a single PZT, and delivered sufficient output speed and load capacity without any need for manual pre-adjustment. Further, the proposed RDP-type actuator was integrated into a designed magnetic resonance imaging (MRI)-compatible microsurgical instrument, which could perform opening and closing motions with a single PZT, and the cutting tests for various test specimens demonstrated its great potential for intraoperative MRI-guided surgery, as shown in Fig. 1.

## Materials and Methods

### Rack-and-pinion inspired RDP and design of RDP-type actuator

**Fig. 2.**
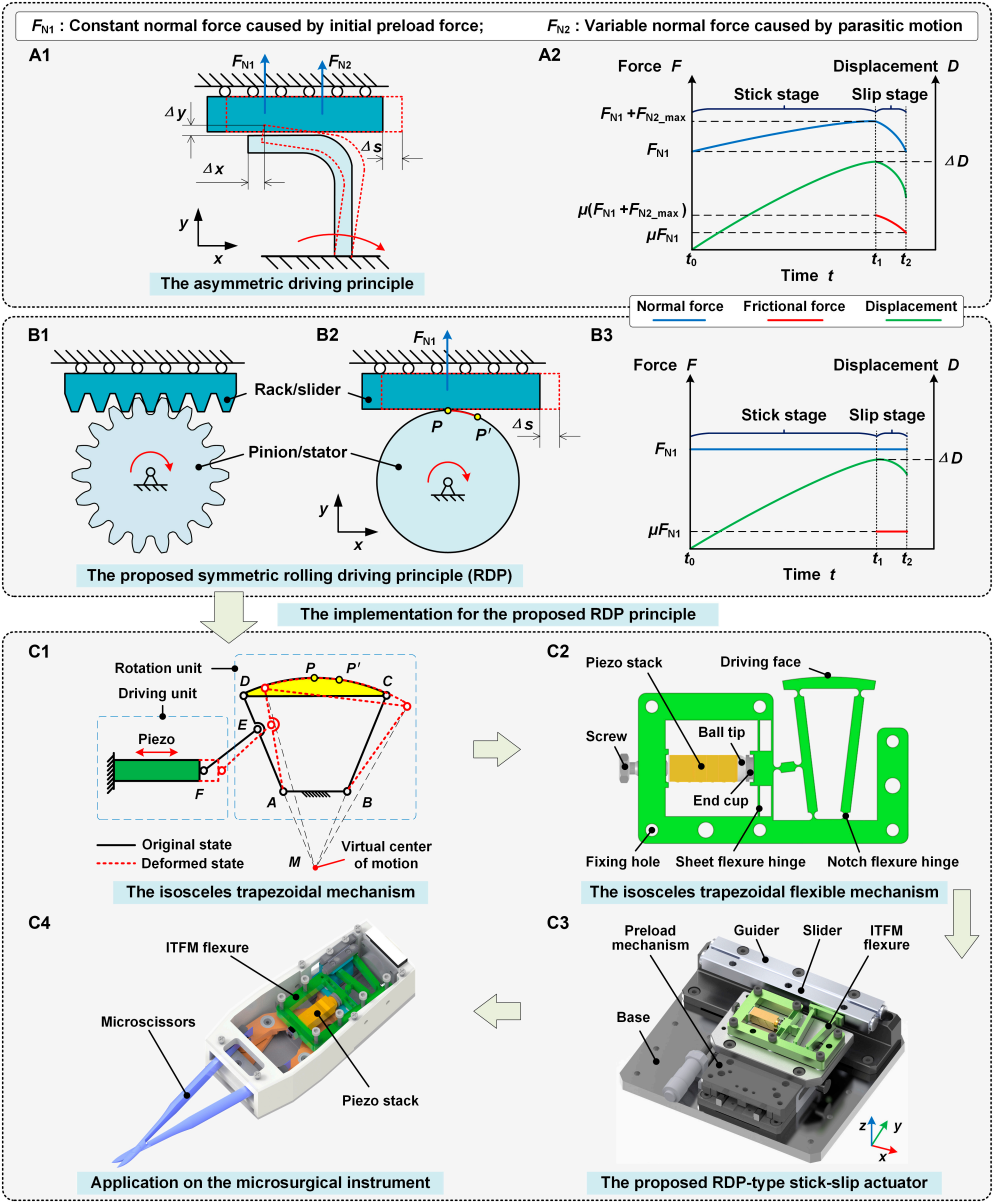
The proposed rack-and-pinion inspired RDP principle and the designed RDP-type stick-slip actuator. (A1) Asymmetric driving principle PMP and its normal force analysis. (A2) Force and motion analysis of the PMP. (B1) Schematic diagram of the rack-and-pinion mechanism. (B2) Schematic diagram of the proposed RDP and its normal force analysis. (B3) Force and motion analysis of the proposed RDP. (C1) Motion model of the isosceles trapezoidal mechanism. (C2) Structural model of the proposed ITFM. (C3) Design overview of the proposed RDP-type actuator. (C4) Designed microsurgical instrument.

The novel RDP is proposed, inspired by the rack-and-pinion mechanism, to provide a simple approach for the design of a linear stick-slip actuator, with excellent performance in bidirectional motion consistency and linearity. The details of the RDP and its comparison with the typical PMP have been illustrated in Fig. 2, with both being analyzed in an ideal stick-slip process. In this process, the stick stage is a pure static friction process with no relative sliding, and the slip stage is a pure sliding friction process with relative sliding only. The PMP and its normal force analysis are shown in Fig. 2A1. As the stator rotates around the fixed end, the driving foot at the end of the stator undergoes a major motion ∆x and a parasitic motion ∆y, driving the slider to produce a stick-slip motion. Its normal force on the slider consists of 2 parts: a constant normal force $F_{N1}$ generated by the initial preload between the stator and the slider, and a variable normal force $F_{N2}$ caused by the varied parasitic motion ∆y. During the forward driving process, the parasitic motion clamps the slider in the stick stage and loosens it in the slip stage, with normal forces changing, as depicted in Fig. 2A2. Conversely, the slider is loosened in the stick stage and clamped in the slip stage in the reverse driving process. The asymmetry driving process challenges the high bidirectional motion consistency achieved by a single PZT [31].

The rack-and-pinion mechanism is capable of achieving high bidirectional motion consistency easily in machine theory. It consists of a guiding rack and a pinion rotating around a fixed axis, as illustrated in Fig. 2B1. The rack can achieve symmetric linear displacement with high consistency due to the symmetric driving structure and contact conditions. Assuming an infinitely small pitch, the rack-and-pinion mechanism can be simplified to be the schematic diagram of the proposed RDP, as shown in Fig. 2B2. For the RDP, the stator is in tangential contact with the slider. As the stator rotates around the fixed center, the driving face $\;{\mathrm{PP}}^{\prime }$ performs pure rolling motion on the surface of the slider and then drives the slider to achieve stick-slip motion. This pure rolling motion keeps the *y*-axis distance between the driving face and the slider constant, making the normal force remain constant to be the initial preload force $F_{N1}$, as shown in Fig. 2B3.

The detailed structural design of the RDP-type stick-slip actuator has been illustrated in Fig. 2C. The corresponding implementation has been designed based on a circular rotation unit that utilizes an isosceles trapezoidal mechanism with 4 links and a driving unit that consists of a piezo stack and a linkage *EF*, as illustrated in Fig. 2C1. The hinge *ABCD* with a circular driving face forms the isosceles trapezoidal mechanism. When the driving piezo stack actuates the link *EF*, the arc *CPD* can move around the virtual center of motion *M* (in case of small deformation), since the point *M* is located at the intersection of the *DA* and *CB* extension lines and serves as the instantaneous rotation center of the arc *CPD*. If the center of the arc *CPD* is designed to coincide with *M*, the arc *CPD*’s motion is a pure rolling motion around *M*. The ITFM flexure has been implemented based on the rigid replacement method to achieve the pure rolling motion of the proposed RDP, as shown in Fig. 2C2. Then, the RDP-type stick-slip actuator is designed based on the core component ITFM, with its configuration illustrated in Fig. 2C2 and C3. This actuator mainly includes a base, a flexure ITFM as a stator (pinion), a linear slider (rack), a preload mechanism to adjust the locking force between the stator and slider, and a piezo stack for driving. The piezo stack is preloaded with a screw fitted with a ball tip and tapered end cup to prevent shear damage from lateral forces. Finally, an MRI-compatible microsurgical instrument integrated with the proposed RDP-type actuator is designed, as shown in Fig. 2C4.

#### Analysis of the novel RDP principle’s advantage

In the proposed RDP principle, the constant normal force of the RDP benefits the reduction of speed fluctuation amplitude and enhances the smoothness of motion, resulting in high motion linearity. This can be proved by the impulse theorem:

$$∆v=v_{\text{stick}}-v_{\text{slip}}=\frac{{\bar{F}}_{\text{slip}}∙t_{\text{slip}}}{m_{\text{slider}}}$$ (1)

$${\bar{F}}_{\text{slip}}=\frac{\int_{0}^{t_{\text{slip}}}f_{\text{slip}}dt}{t_{\text{slip}}}$$ (2)

where ∆v denotes the speed variation; $v_{\text{stick}}$ and $v_{\text{slip}}$ are the speeds at both ends of the stick and slip stage; and ${\bar{F}}_{\text{slip}}$ and $f_{\text{slip}}$ are the mean and real-time value of the sliding friction force in the slip stage. The constant variables $m_{\text{slider}}$ and $t_{\text{slip}}$ express the slider’s mass and the slip stage’s duration, respectively. Hence, a smaller ${\bar{F}}_{\text{slip}}$ of the proposed actuator will provide less speed variation. Under the same initial preload $F_{N1}$ and forward motion ∆D in the stick stage, the proposed rack-and-pinion inspired RDP exhibits a smaller sliding friction ${\bar{F}}_{\text{slip}}={\mu F}_{N1}$, compared to the PMP whose sliding friction force decreased from $\mu \left(F_{N1}+F_{N2\_\max}\right)$ to ${\mu F}_{N1}$ (μ is the sliding friction coefficient), as shown in Fig. 2A2 and B3. Therefore, the proposed rack-and-pin-ion inspired RDP has the potential to produce higher motion linearity due to constant normal force and less speed variation.

**Fig. 3.**
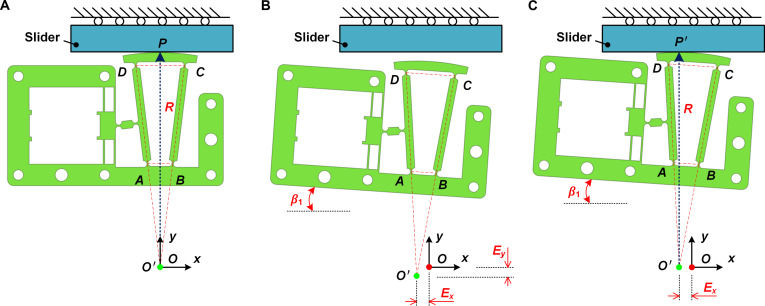
Analysis of the proposed RDP principle’s self-adaptability to assembly errors. (A) Ideal assembly status. (B) Actual assembly status and its associated position and angular errors. (C) Actual assembly status after compensation by the preload mechanism.

Furthermore, the stator’s arc-shaped driving surface, with its center coinciding with the stator’s rotational center, maintains tangential contact with the mover. This geometric configuration ensures identical forward and reverse driving processes and enables insensitivity and self-adaptation to assembly errors. Thus, no manual pre-adjustment processes are required for improving bidirectional consistency. The mentioned insensitivity and self-adaptation have been further explained in Fig. 3, with the proposed RDP-type actuator serving as a case to demonstrate the self-adaptive capability. The ideal assembly status is illustrated in Fig. 3A. The intersection of the *DA* and *CB* extension lines coincides with the fixed point *O*, and the line *AB* of the ITFM is parallel to the *x* axis. The actual assembly status and errors are enlarged for better illustration, as shown in Fig. 3B. *E_x_* and *E_y_* denote the position errors after assembly in the *x* and *y* directions, respectively. *β*_1_ stands for the angular errors after assembly. For most linear stick-slip actuators, the position errors in the *x* direction generally do not affect the output performance, and the position errors in the *y* direction can be easily compensated by the preload mechanism, as shown in Fig. 3C. In contrast, the angular error is the primary factor hindering the previous studies from achieving high bidirectional motion consistency. In the presented design, this angular error does not affect the RDP-type actuator’s bidirectional motion consistency due to the identical tangential contact condition in Fig. 3A and C. This identical tangential contact condition can be well maintained as points *P* and *P′* both undergo circular motion with radius *R* around a fixed center, although the angular error *β*_1_ and position error *E_x_* remain, as illustrated in Fig. 3C. Hence, the RDP is self-adaptive to assembly errors.

#### Operating principle of the proposed RDP-type actuator

**Fig. 4.**
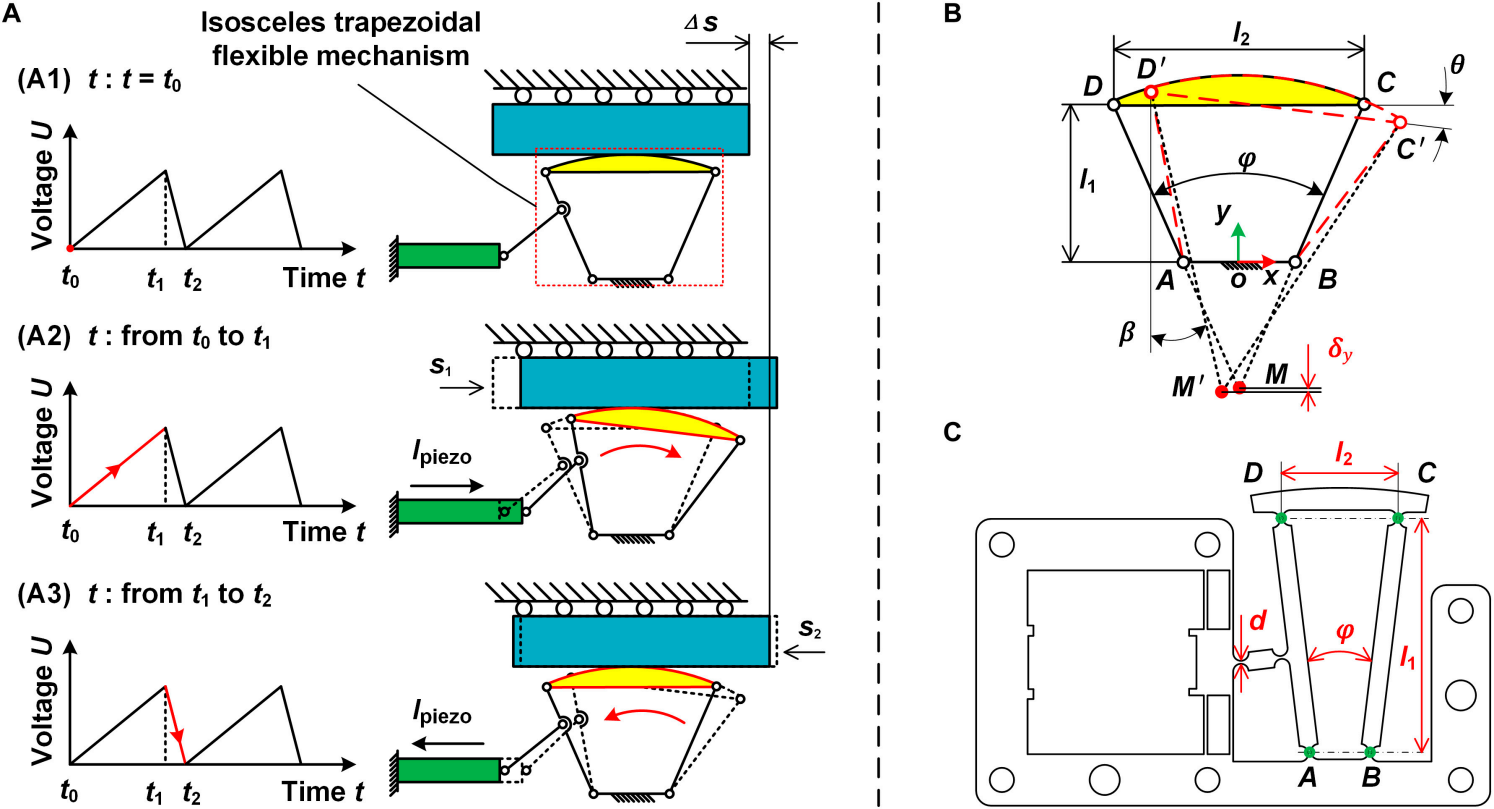
Operation process of the proposed actuator and the axial drift and dimension of the ITFM. (A) Operation process of the proposed actuator: (A1) Initial state. (A2) Stick stage. (A3) Slip stage. (B) ITFM’s axial drift. (C) ITFM’s structural parameters (unit: mm).

The operating principle of the proposed RDP-type actuator is illustrated in Fig. 4A. During the forward driving process, the voltage rises gradually and then falls rapidly within a driving cycle. The piezo stack repeats this cycle of slow elongation and rapid shortening under continuous excitation, causing the slider to move along the *x* axis. The entire operating cycle includes the following stages: the initial state at time $t_{0}$, stick stage during [$t_{0}$, $t_{1}$], and slip stage during [$t_{1},t_{2}$].

At the initial state (Fig. 4A1): The piezo stack is at the original state since the excitation voltage is zero, with an initial preload force applied by the preload mechanism to keep the slider and the circular driving face firmly in contact. During the stick stage (Fig. 4A2): The amplitude of the excitation voltage rises slowly with time, drives the piezo stack to elongate, and pushes the circular driving face of the flexure ITFM to rotate clockwise around the virtual center of rotation slowly. Such slow clockwise rotation generates a positive static friction force on the slider, driving it to produce a forward linear displacement $s_{1}$ along the *x* axis. During the slip stage (Fig. 4A3): The excitation voltage’s amplitude rapidly drops to zero, making the piezo stack contract quickly to its original state. The flexure ITFM returns to its initial state due to the motion of elastic recovery. This recovery process makes the circular driving face rotate counterclockwise rapidly, producing an opposing sliding friction force. Then, a slight reverse linear displacement $s_{2}$ along the *x* axis is generated under the combined effect of sliding friction force and the inertia force of the slider. The net displacement of the slider in an entire operating cycle is $∆s=s_{1}-s_{2}$, with a backward ratio $b_{r}=s_{2}/s_{1}$. Hence, an infinitely long stroke can be achieved in theory by repeating the operating process.

### Actuator’s design optimization based on finite element method

To improve the performance of the proposed RDP-type actuator, the axis drift of the ITFM flexure has been defined and modeled, and design optimization has been conducted to investigate the optimal structural variables of the ITFM flexure based on the finite element method (FEM) via ANSYS Workbench 20.0.

#### Definition and modeling of the ITFM’s axis drift

Axial drift directly affects the ITFM mechanism’s ability to generate pure rolling motion, thus requiring detailed analysis. As shown in Fig. 4B, the axial drift phenomenon is enlarged for better illustration. The link *CD* is considered a rigid body with a fixed rotation point *M* attached to it. When the link *CD* moves to position *C'D'*, the center of rotation shifts to *M'*, and the displacement deviation of point *M* is defined as the axial drift δ.

The axial drift δ can be modeled by the movements of points *C* and *D*: $∆C_{x},∆C_{y},∆D_{x},∆D_{y}$, and the parameters $l_{1}$, φ, and $l_{2}$ donate the 3 structural parameters of the long side’s height, angle, and length for the trapezoid *ABCD*. With the coordinate system established at the midpoint of link *AB*, it can be expressed as follows:

δx=Mx′−Mxδy=My′−My (3)

and the coordinate of points *M* and *M'* can be expressed as:

$$\left\{\begin{array}{c} M=\left(M_{x},M_{y}\right)=\left(0,l_{1}-\frac{l_{2}}{2\text{tan}\left(0.5\varphi \right)}\right)\\ M^{\prime }=\left(M_{x}^{\prime },M_{y}^{\prime }\right)=\left(D_{x}^{\prime }+R_{a}\text{sin}\beta ,D_{y}^{\prime }-R_{a}\text{cosβ}\right) \end{array}\right.$$ (4)

where $R_{a}$ and β denote the radius of the arc-shaped driving surface and the angle between line *DM* and the *y* axis:

$$R_{a}=l_{\mathrm{DM}}=\frac{l_{2}}{2\text{sin}\left(0.5\varphi \right)}$$ (5)

β=0.5φ−θ (6)

where θ denotes the angle between line *CD* and the *x* axis. It can be calculated by the coordinate of points *C*, *D*, *C′*, and *D′*:

$$\theta =\text{arccos}\left(\frac{D^{\prime }C^{\prime }∙DC}{\left|D^{\prime }C^{\prime }\right|\left|DC\right|}\right)$$ (7)

C=0.5l2l1D=−0.5l2l1C′=Cx+∆CxCy+∆CyD′=Dx+∆DxDy+∆Dy (8)

#### FEM-based design optimization

The primary structural variables that influence the ITFM’s performance are selected and labeled in Fig. 4C. Among them, d donates the thickness of the notch flexure hinge, and $l_{1}$, φ, and $l_{2}$ have been expressed above. The overall length, width, and height dimensions of the ITFM are 70 mm × 43.3 mm × 7.2 mm. The width of the installing groove for the piezo stack is selected as the same value of 7.2 mm to suit the selected piezo stack. The length and thickness of the sheet flexure hinge are determined to be 8 and 0.5 mm, respectively, to ensure a high length–thickness ratio and improved hinge performance. The fabrication material AL7075 has been selected owing to its elasticity and lightweight. Its elastic modulus, Poisson ratio, and density values are 7.17 × 10^4^ MPa, 0.33, and 2,810 kg/m^3^, respectively.

The *y* component of this drift is selected as the optimization objective since it is the primary factor affecting the ability to achieve the RDP. Hence, the objective function *Z* is defined as the minimization of the drift’s *y*-component amplitude. The following mathematical statement can describe this optimization problem:

$$Z=\text{Min}\;\left|\delta_{y}\left(X\right)\right|,\;X={\left[d,\;l_{1},\;\varphi ,\;l_{2}\right]}^{T}$$ (9)

subject to0.4mm≤d≤0.7mm30.5mm≤l1≤33.0mm12°≤φ≤15.5°14.0mm≤l2≤18.0mm (10)

where $\left|\delta_{y}\left(X\right)\right|$ represents the drift’s *y*-component amplitude of the ITFM. It is calculated by the simulation result of $∆C_{x},∆C_{y},∆D_{x},∆D_{y}$. ***X*** is defined to donate the vector of the structural variables ${\left[d,\;l_{1},\;\varphi ,\;l_{2}\right]}^{T}$. The variable variation ranges have been defined as a set of constraint functions based on machining capacity and mechanism configuration.

**Fig. 5.**
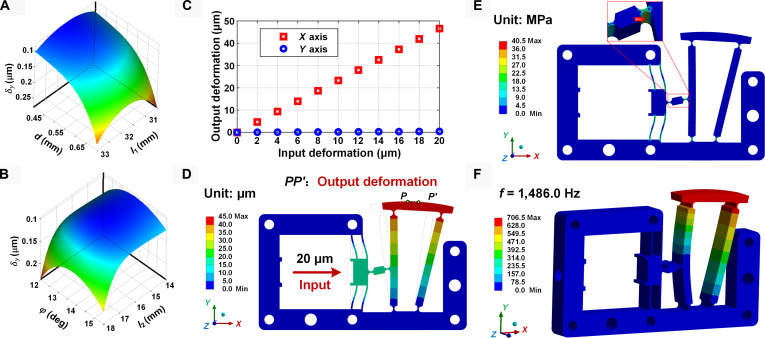
FEM-based simulation for the ITFM flexure. (A and B) Response surface of constraint functions with respect to the structural variables. (C) Desired output displacement by FEA. (D) Deformation behaviors. (E) Stress distribution. (F) First modal shape of the proposed ITFM.

The simulated response surfaces reveal the relationship between the drift’s *y*-component amplitude and the structural variables, as shown in Fig. 5A and B. $\delta_{y}\left(X\right)$ reduces with the decrease of d and first decreases and then increases with the increase of $l_{1}$, φ, and $l_{2}$. For better illustration, the positive direction of the $\delta_{y}$ axis is oriented downward. Subsequently, the optimal results are derived utilizing the genetic algorithm. The detailed values before and after design optimization are summarized in Table 1. The *y*-component amplitude of the axial drift demonstrates a noticeable reduction of 30.51%.

**Table 1.** Optimization results

Parameters	Initial value	Optimal value
*d*/mm	0.5	0.4
*l*_1_/mm	32.2	31.8
*φ*/deg	14.2	13.8
*l*_2_/mm	15	15.9
*δ_y_*/nm	126.2	87.7

### Investigation of the ITFM’s static and dynamic performances

The static and dynamic performances of the optimized flexure ITFM are analyzed, as shown in Fig. 5C to F. The relationship between the input and output displacement has been investigated, as displayed in Fig. 5C. The *x*-axis output displacement increases linearly with the increasing input displacement. In contrast, the *y*-axis displacement is almost unaffected and remains constant at around 0. This result demonstrates the ITFM flexure’s superior ability to implement the RDP. The maximum *x*-direction deformation of 46.65 μm appears at the driving face of the ITFM, as shown in Fig. 5D. The maximum equivalent stress of 40.497 MPa occurs at the flexure joint between the connecting rod and the isosceles trapezoidal module, as shown in Fig. 5E. It is less than the allowable stress of 505 MPa for the selected material. The modal analysis shows that the first response frequency of the ITFM is 1,486.0 Hz (Fig. 5F).

## Results

### Experimental setup for performance investigation

**Fig. 6.**
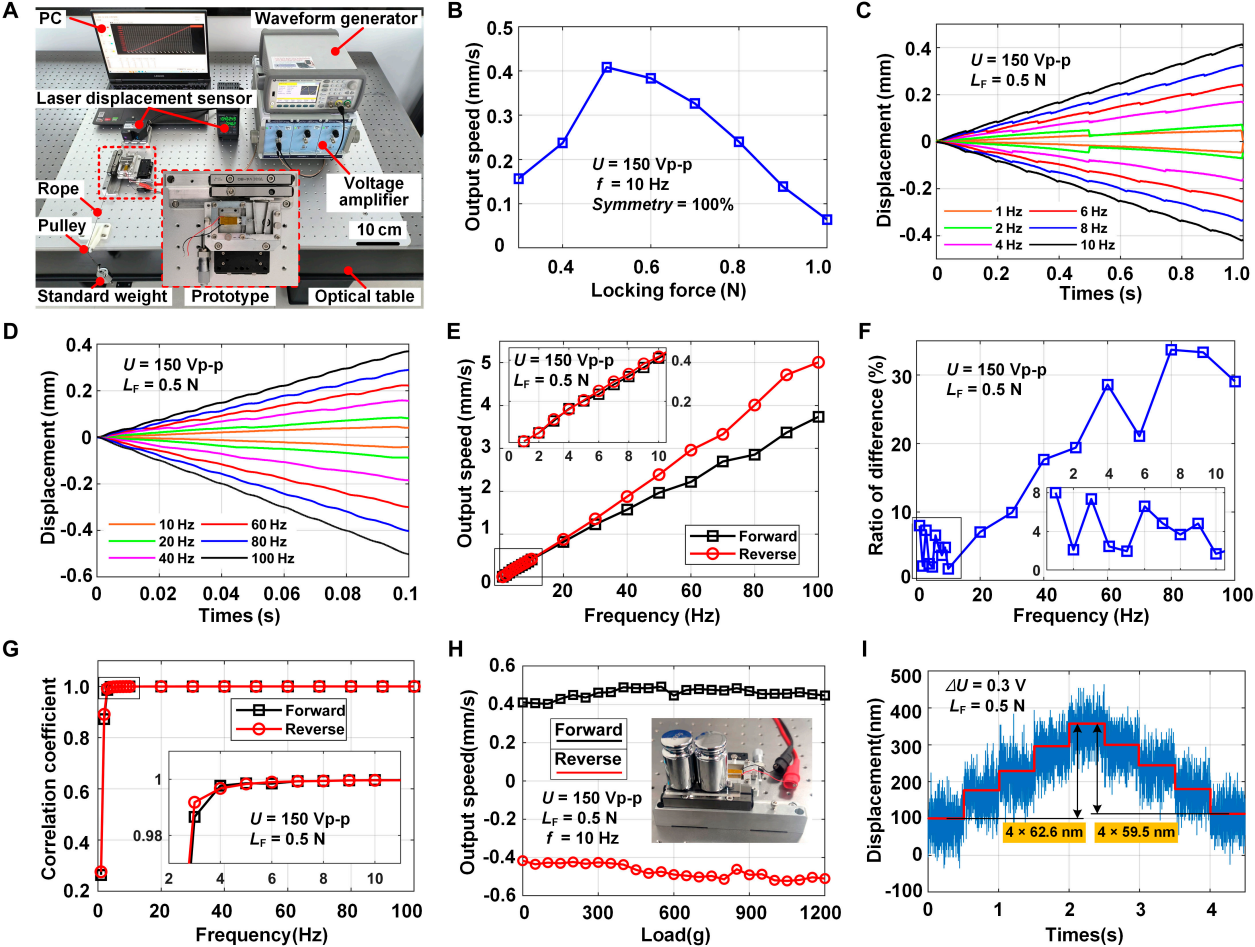
The established experimental system and the test of the proposed actuator under low-frequency conditions. (A) Hardware configuration of the experimental system. (B) Relationship between the output speed and the locking force. (C) Displacement curve under [1 Hz, 10 Hz]. (D) Displacement curve under [10 Hz, 100 Hz]. (E) Relationship between the bidirectional output speed and the driving frequency under [1 Hz, 100 Hz]. (F) Relationship between the velocity difference ratio and the driving frequency. (G) Relationship between the linear correlation coefficient and the driving frequency. (H) Load capacity under low frequency. (I) Motion resolution under the locking force of 0.5 N.

To investigate the performance of the prototyped design, the experimental setup has been configured on a vibration-isolated optical table, as illustrated in Fig. 6A. The experimental system integrated multiple instruments: a prototyped stick-slip actuator, a waveform generator (33500B, Keysight, USA), a voltage amplifier (E01.A3, Coremorrow, China), a laser displacement sensor (LK-H050, Keyence, Japan), and a PC. The piezo stack PK4GQP1 (7 mm × 7 mm × 18 mm, 0 to 150 V, 20 μm, 3.0 μF, Thorlabs, USA) has been selected to drive the ITFM flexure and the cross-roller linear guide (VR4-80, 62 g, THK, Japan). The overall size of the prototyped RDP-type actuator is 140 mm × 110 mm × 34 mm. Linear stick-slip motion was achieved by applying a sawtooth signal from the waveform generator, which underwent 15-fold amplification before driving the prototype actuator. The output motion was collected by the laser displacement sensor and recorded by the PC. Besides, a pulley-rope-weight unit has been designed to quantify the magnitude of the locking force between the flexure and the slider. The proximal end of the rope connects with the slider, and the other end hangs the standard weight. The locking force is defined as the weight suspended at the end of the rope when the slider shows a sliding tendency during the adjustment of the preload mechanism.

### Output performances under low frequency

A series of experiments was first performed under low driving frequency to investigate the actuator’s potential for low-speed, high-precision applications. The output displacements and speeds were initially tested to assess the characteristics of bidirectional motion consistency, and the bidirectional motion linearity was analyzed to confirm the actuator’s capacity for achieving smooth motion. Finally, further investigation of the bidirectional load capacity and resolution was carried out.

#### Determination of locking force for low-frequency tests

The driving sawtooth wave with an excitation voltage of 150 Vp-p and symmetry of 100% has been applied, with the locking force of [0.5 N, 3.5 N] adjusted by the weights. The output speed of the slider was tested at 10 Hz. The relationship between the locking force and output speed is illustrated in Fig. 6B. The output speed tends to increase and then decrease with the increasing locking force, and the output speed values peak at the locking force of 0.5 N. Therefore, 0.5 N is selected as the optimal locking force for the low-frequency tests.

#### Bidirectional motion consistency

Under this optimal locking force, the driving sawtooth wave with an excitation voltage of 150 Vp-p and symmetry of 100% and 0% has been applied for forward and reverse motion, respectively. The slider’s displacement was tested under [1 Hz, 100 Hz], and the relationship between the driving frequency and output displacement is illustrated in Fig. 6C and D. The forward and reverse displacement curves presented excellent symmetry within [1 Hz, 20 Hz]. However, the deviation of displacement becomes more marked as the frequency rises from [20 Hz, 100 Hz]. This increasing deviation is attributed to the more marked accumulation of differences in step displacement as frequency rises [32]. The experimental results indicate that the bidirectional step displacements for forward and reverse motion are 41.0 and 41.7 μm at 10 Hz, as well as 37.36 and 50.06 μm at 100 Hz, respectively.

Furthermore, the output speed of the slider was tested, and the driving frequency versus output speed curve is shown in Fig. 6E. The output speed increases with higher driving frequency due to the increased number of cycles per unit of time. Similarly, the forward and reverse output speeds matched well within [1 Hz, 20 Hz], showing high bidirectional motion consistency. However, the deviation of the bidirectional output speeds increases as the driving frequency increases from [20 Hz, 100 Hz]. This may be attributed to the unstable contact between the slider and stator, resulting from excessive vibration as the frequency rises under the low locking force of 0.5 N [31]. These results show that the bidirectional output speed values are 0.410 and 0.417 mm/s at 10 Hz, and 3.736 and 5.006 mm/s at 100 Hz.

To depict the bidirectional motion consistency, the velocity difference ratio λ has been proposed:

$$\lambda =\frac{2\left|v_{F}-v_{R}\right|}{\left(v_{F}+v_{R}\right)}\times 100\%$$ (11)

where $v_{F}$ and $v_{R}$ represent the actuator’s mean output speed in forward and reverse motions under identical conditions, respectively. The velocity difference ratio under [1 Hz, 100 Hz] was calculated according to Eq. (11), and the relationship between the velocity difference ratio and the driving frequency is shown in Fig. 6F. The results demonstrated that within the [1 Hz, 20 Hz] frequency range, the actuator achieved superior bidirectional motion consistency, maintaining velocity variations below 10%. However, operational consistency deteriorated substantially at frequencies exceeding 20 Hz. The minimum velocity difference ratio of 1.96% occurs at the driving frequency of 10 Hz, showing an excellent bidirectional consistency performance of the proposed RDP-type actuator.

#### Bidirectional motion linearity

The displacement curves in Fig. 6C and D indicate that the actuator generates output motions with strong linearity at wide bandwidths, with decreased or eliminated backward motions. Then, further investigation was performed on the actuator’s ability to produce smooth motion. These displacement curves were fitted linearly within [1 Hz, 100 Hz], and the resulting linear correlation coefficient $R^{2}$ was calculated to assess the linearity of the displacements. The closer the $R^{2}$ to 1, the better the linearity and smoothness of the output motion. The relationship between driving frequency and the linear correlation coefficient $R^{2}$ is shown in Fig. 6G. $R^{2}$ increases as the driving frequency rises and approaches 1 under [4 Hz, 100 Hz], indicating a high linear displacement output capability. However, the linearity is poor under [1 Hz, 3 Hz], because the frequency is too low and the actuator is operated in stick–stick mode [37]. The bidirectional linear correlation coefficients for forward and reverse motion are 0.99969 and 0.99962 at 10 Hz, as well as 0.99999 and 0.99999 at 100 Hz, respectively. These results verify that the actuator can produce smooth motion with high motion linearity for bidirectional motion under low frequencies.

#### Bidirectional load capacity

The vertical load capacity was tested for bidirectional motion considering the practical applications, under the driving frequency of 10 Hz. A weight plate that carried standard weights was placed on the slider with a total weight of 74 g for vertical loading, as shown in the middle of Fig. 6H. The slider output speed was tested under different vertical loads from 0 to 1,200 g, as depicted in Fig. 6H, since the plate cannot take on any more weight. The bidirectional output speed appears to remain stable around 0.45 mm/s despite the increase of vertical load, showing strong insensitivity to the load. This merit can be attributed to the actuator’s maximum output power, which is sufficiently high to keep the output speed unaffected by an increase in the vertical load. The experimental results indicate that under a frequency of 10 Hz and a vertical load of 1,200 g, its bidirectional output speeds are 0.446 and 0.510 mm/s.

#### Bidirectional motion resolution

The bidirectional motion resolution of the prototyped actuator was measured under the locking force of 0.5 N. A stepping signal that increased from 0 to 1.2 V and then decreased to 0 V with a step of 0.3 V and lasted 4.5 s was applied to drive the piezo stack. The response displacement of the actuator was measured in Fig. 6I, and the bidirectional resolutions are determined as 62.6 and 59.5 nm.

### Output performances under high frequency

To investigate the stick-slip actuator’s performance for high-speed, high-load applications, further performance tests were carried out at high driving frequencies. The effect of driving frequency was first investigated to determine the maximum output speed, bidirectional motion consistency, and motion linearity. Then, the proposed speed fluctuation coefficient was tested. Finally, the capacity of vertical loading and the resolution were tested and measured for bidirectional motion.

#### Determination of locking force for high-frequency tests

**Fig. 7.**
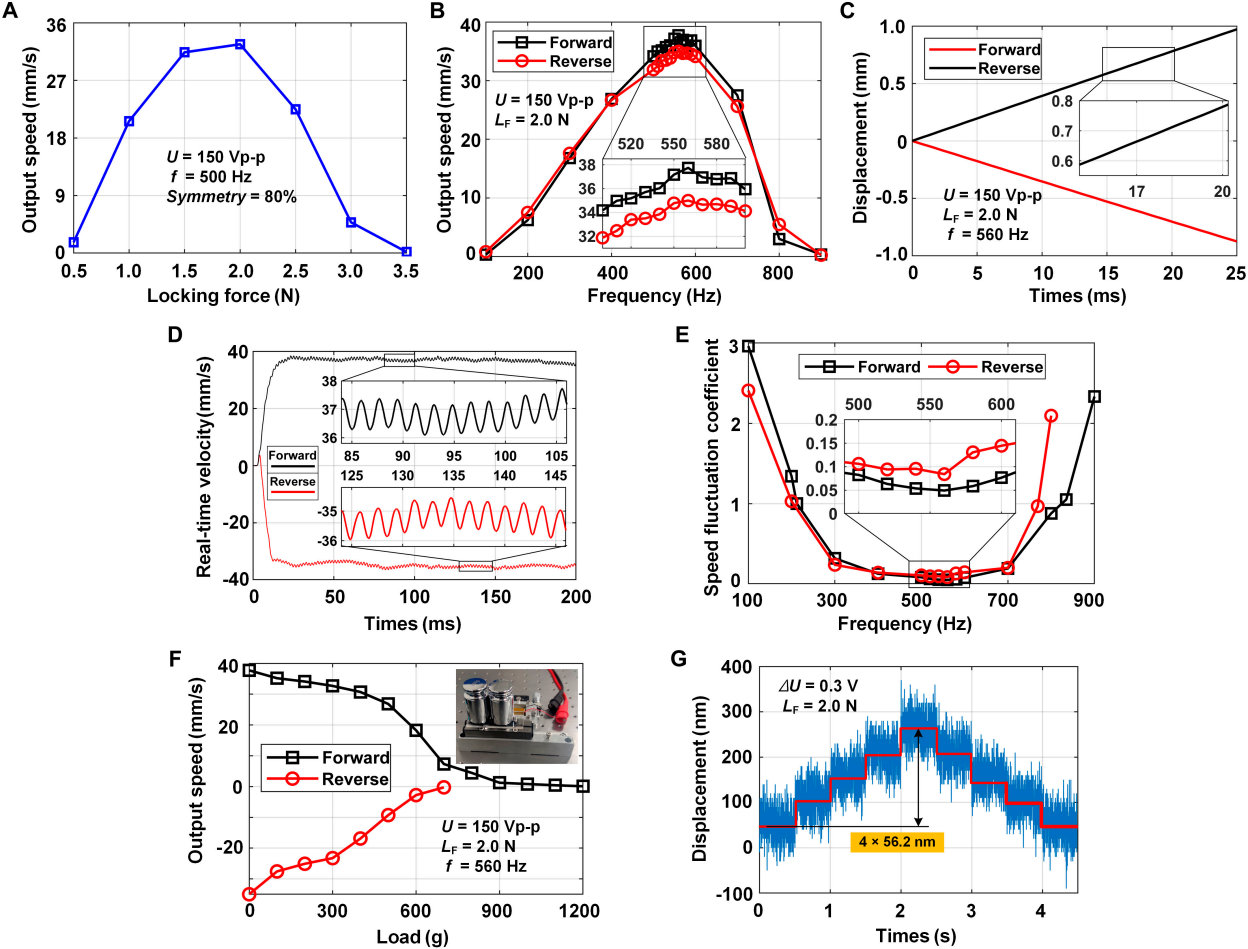
Performance investigation of the proposed actuator under high-frequency conditions. (A) Relationship between the output speed and the locking force. (B) Relationship between the bidirectional output speed and the driving frequency under [100 Hz, 900 Hz]. (C and D) Displacement and real-time velocity of the slider at 560 Hz. (E) Relationship between the speed fluctuation coefficient and the driving frequency. (F) Load capacity under high frequency. (G) Motion resolution under the locking force of 2.0 N.

Similarly, the optimal locking force at high frequency was tested, with the driving sawtooth wave configured with an excitation voltage of 150 Vp-p and 80% symmetry. The driving frequency was set to 500 Hz, as the minimum charge–discharge time for the selected piezo stack and voltage amplifier was estimated to be 0.4 ms. The relationship between locking force and output speed is illustrated in Fig. 7A. The results show that the output speed values peak at the locking force of 2.0 N, which was consequently selected as the optimal locking force for high-frequency tests.

#### Bidirectional output speed and motion linearity

Under the locking force of 2.0 N, the driving sawtooth wave with an excitation voltage of 150 Vp-p, a forward motion symmetry of 80%, and a reverse motion symmetry of 20% has been applied. During [100 Hz, 900 Hz], the relationship between the driving frequency and the bidirectional output speed of the slider was investigated, as illustrated in Fig. 7B. The output speed increases at first and then decreases as the driving frequency increases. The reason for this phenomenon is that the actuator achieves the ultimate response time when the driving frequency reaches a threshold; once exceeding this threshold, the response time becomes too short for the ITFM to elongate and contract sufficiently. The results show that the bidirectional output speed values peak at the same driving frequency of 560 Hz, with the maximum forward and reverse output speed of 37.73 and 34.99 mm/s, showing a high bidirectional motion consistency with a velocity difference ratio of 7.54%.

In order to further investigate the motion linearity at high frequencies, the bidirectional slider’s displacement was tested at the symmetry of 80% and 20% under various driving frequencies. Take 560 Hz for instance, as shown in Fig. 7C. The backward motion disappeared in both forward and reverse driving directions, and even the linear motions appeared since stable continuous forward movements were produced in the slip stages. The results indicate that the backward ratio values are −98.8% and −98.2% for forward and reverse motions, respectively. The fitting results demonstrate great motion linearity, with the bidirectional linear correlation coefficients $R^{2}$ to be 0.99999 and 0.99999 at 560 Hz.

These continuous forward movements during the slip stage have also appeared occasionally in the reported works [38]. It can be explained by the impulse theorem, as Eqs. (1) and (2) described in Materials and Methods. ∆v in slip stage is constant for a given locking force, so $v_{\text{slip}}$ can be positive as the lower ∆v of the RDP-type actuator, leading to the forward movements in the slip stage. For further verification, the bidirectional real-time velocity of the slider was tested at 560 Hz, as shown in Fig. 7D. The bidirectional velocities fluctuate slightly and periodically around the average output speed, with the moving directions remaining unchanged in both the stick and slip stages. Hence, the proposed RDP-type actuator can achieve nearly linear bidirectional motion without motion direction change, strongly verifying the advantage of achieving smooth motion with high motion linearity under high driving frequencies.

#### Definition and investigation of the proposed speed fluctuation coefficient

The speed fluctuation coefficient ε was proposed, because the backward ratio and motion linearity calculated from the displacement data provide a less intuitive description of the smoothness of motion. ε was obtained based on the velocity data, as Eq. (12) described:

$$\varepsilon =\frac{v_{\text{max}\_\text{stick}}-v_{\text{min}\_\text{slip}}}{v_{\text{max}\_\text{stick}}}$$ (12)

where $v_{\max\_\text{stick}}$ denotes the maximum vector speed of the stick stage and $v_{\min\_\text{slip}}$ is the minimum vector speed of the slip stage. It can reflect the fluctuation magnitude of stick-slip motion fully and directly in the summarized 3 cases of backward motion: (1) the backward motion is noticeable; (2) the backward motion is eliminated; and (3) the continuous forward motion is produced. For case (1), the backward ratio is positive ($v_{\min\_\text{slip}}<0$), the backward motion remains, and ε>1. A larger ε indicates a stronger motion fluctuation with the frequent motion direction change. For case (2), the backward ratio is 0 ($v_{\min\_\text{slip}}=0$), the backward motion is completely eliminated, and thus ε=1. For case (3), the backward ratio is negative ($v_{\min\_\text{slip}}>0$), the continuous forward motion appears, and 0≤ε<1. A smaller ε indicates a smoother motion with tinier fluctuation.

The bidirectional real-time velocity of the slider was tested under [100 Hz, 900 Hz], and ε was calculated using the upper and lower bounds of the speed fluctuation after stabilization. The relationship between the driving frequency and the speed fluctuation coefficient was investigated, as shown in Fig. 7E. For the forward driving process, the actuator’s speed fluctuation coefficient is less than 1.0 in the frequency range of [212 Hz, 835 Hz], and its minimum value of 0.0494 is obtained at 560 Hz. The range for the reverse driving process is [200 Hz, 769 Hz], and its minimum value is 0.0842 at 560 Hz. Hence, this actuator can achieve smooth bidirectional motion in the bandwidth of [212 Hz, 769 Hz].

#### Bidirectional load capacity

The vertical load capacity under high frequency was tested in the same way as the experiment carried out in low frequency. With the driving frequency of 560 Hz, the slider output speed was tested as the vertical loads increased from 0 to 1.2 kg, as depicted in Fig. 7F. It can be seen that the output speed decreased with increasing load due to the increased equivalent inertia of the slider. The maximum vertical loads are 1,200 and 700 g for the forward and reverse driving processes, respectively.

#### Bidirectional motion resolution

The motion resolution of the prototyped actuator was measured again under the locking force of 2.0 N with the same stepping signal applied to drive the piezo stack. The actuator’s response displacement is shown in Fig. 7G, and the resolution is determined as 56.2 nm.

### Application in MRI-compatible microsurgical instrument

**Fig. 8.**
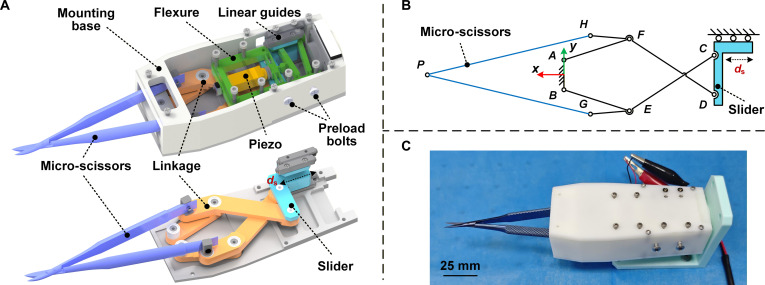
The detailed design and the prototype of the stick-slip actuator-based microsurgical instrument. (A) Designed microsurgical instrument. (B) Linkage of the microsurgical instrument. (C) Prototyped microsurgical instrument.

To validate the practical application potential of the proposed RDP-type stick-slip actuator, the actuator was integrated into a specifically designed microsurgical instrument, as illustrated in Fig. 8. The surgical instrument comprises a mounting base, an ITFM flexure, a slider, a piezo, micro-scissors, a linkage mechanism, and dual cross-roller linear guides. As shown in Fig. 8A and B, the linkage mechanism incorporates rod *AB* as the fixed link, with the 2 components of the micro-scissors fixed to hinges *G* and *H*, respectively. The dual cross-roller linear guides connect the slider to the mounting base, facilitating its movement along the *x* axis to produce the opening and closing motion of the micro-scissors. The ITFM flexure, mounted on the base, maintains firm contact with the slider, while the contact force can be adjusted through preload bolts. The operational workflow proceeds as follows: Under sawtooth wave excitation, the piezo undergoes periodic extension and contraction, causing the ITFM flexure to deform and drive the slider in a stick-slip motion. When the slider moves in the positive *x*-axis direction, the micro-scissors close; conversely, they open when moving in the negative *x*-axis direction. The integrated surgical instrument, as shown in Fig. 8C, is constructed entirely from MRI-compatible materials, including resin, aluminum alloy, titanium alloy, and stainless steel. Combined with the piezo’s inherent resistance to electromagnetic interference, this design demonstrates great potential for surgical applications in MRI environments.

**Fig. 9.**
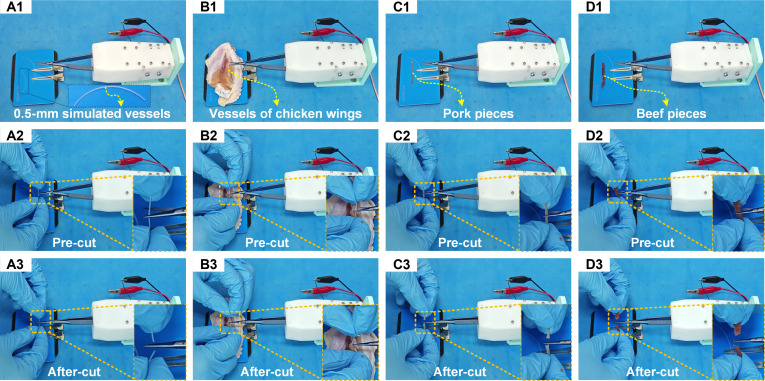
The cutting experiments of the prototyped microsurgical instrument. (A1-A3) The 0.5-mm simulated vessels. (B1-B3) Ex vivo chicken wing blood vessels. (C1-C3) Pork pieces. (D1-D3) Beef pieces.

Further, experiments were conducted with the surgical instrument prototype to evaluate its overall performance under open-loop conditions. Initially, opening and closing motion tests were performed. Under excitation signals with a symmetry of 10% duty cycle, the micro-scissors completed an opening motion within 2 s; while under a symmetry of 90%, the closing motion was achieved in 2 s, validating the designed instrument’s capability to execute opening and closing movements. Subsequently, to verify the cutting performance, various test specimens were prepared, including 0.5-mm simulated vessels, ex vivo chicken wing blood vessels, pork pieces, and beef pieces. Under excitation signals with a symmetry of 90%, the microscissors successfully performed closing motions and effectively severed all prepared specimens, demonstrating excellent cutting capabilities, as shown in Fig. 9. The opening–closing motions and specimen cutting processes were documented through video recordings, with detailed demonstrations provided in the attached video.

## Discussion

Table 2 compares the proposed RDP-type actuator and state-of-the-art designs regarding the number of PZT, velocity difference ratio, adjustment difficulty for keeping consistency, motion linearity, speed fluctuation, output speed, and size of the actuator. The best results of these designs during their experiments are selected and listed in this table for comparison. The presented actuator can perform smooth bidirectional motion under both low and high frequencies with one single PZT stack due to the symmetric driving structure, while most other designs utilize 2 or more PZTs. This actuator produces a velocity difference ratio of 1.96% in bidirectional motion at 10 Hz and remains to be 7.54% at 560 Hz, indicating that it achieves a high bidirectional motion consistency and is comparable with the best results of the others. Furthermore, no manual pre-adjustment processes are required. The bidirectional motion linearity can reach 0.99969 and 0.99962 under 10 Hz, as well as 0.99999 and 0.99999 under 560 Hz, showing its capacity for achieving high bidirectional motion linearity. In contrast, the other designs have no investigation into this issue or only achieved high motion linearity in a single direction. The speed fluctuation coefficient has been defined to describe the smoothness of stick-slip motion in the velocity stage. The smaller value of the speed fluctuation coefficient indicates a smoother output motion. The proposed design achieves the smallest speed fluctuation coefficient of 0.0494 under 560 Hz, while most other designs are greater than or equal to 1.0. Thus, the proposed design could generate smooth output motion with tiny speed fluctuation at high driving frequencies. Besides, our design achieves an excellent output speed of 0.410 and 37.73 mm/s under 10 and 560 Hz, respectively, with a comparable size to other studies. Therefore, the presented RDP-type stick-slip actuator achieves bidirectional smooth motion with high consistency and linearity, and yields excellent performance in most listed items with no manual pre-adjustment processes. This actuator also demonstrates a high potential to fulfill the needs of different applications under both low and high frequencies. For various applications, a simple adjustment is required to adjust the locking force for the proposed design, because a high locking force is necessary to ensure the stick-slip motion mode under high driving frequency.

**Table 2.** Comparisons with state-of-the-art works

	This work	Ref. [30]	Ref. [31]	Ref. [32]	Ref. [36]	Ref. [29]	Ref. [41]
Number of PZT	Single-PZT	Four-PZT	Two-PZT	Two-PZT	Two-PZT	Four-PZT	Single-PZT
Velocity difference ratio	1.96% (150 V,10 Hz) 7.54% (150 V, 560 Hz)	18.5% (120 V, 1 Hz)	1.6% (100 V, 10 Hz)	6.8% (100 V, 10 Hz)	NG	NG	NG
Adjustment difficulty for keeping consistency	Need no adjustment	Hard	Hard	Easy	NG	NG	NG
Motion linearity	0.99969 and 0.99962 (10 Hz, high) 0.99999 and 0.99999 (560 Hz, high)	NG (low)	NG (low)	NG (low)	NG (middle)	NG (middle)	0.9999 (10 and 1,300 Hz, high)
Speed fluctuation coefficient	FM: 0.0494 (560 Hz) RM: 0.0842 (560 Hz)	>1.0 (8 Hz)	>1.0 (100 Hz)	>1.0 (10 Hz)	~1.0 (25 Hz)	~1.0 (20 Hz)	<1.0 (10 and 1,300 Hz)
Output speed/(mm·s^-1^)	0.410 (10 Hz) 37.73 (560 Hz)	0.0046 (8 Hz)	0.23 (100 Hz)	0.199 (10 Hz)	<0.18 (25 Hz)	<0.08 (20 Hz)	0.114 (10 Hz) 101.76 (2,000 Hz)
Size/mm^3^	140 × 110 × 34	140 × 100 × 20	NG	118 × 52 × 38	NG	NG	75 × 47.5 × 10

NG, not given; FM, forward motion; RM, reverse motion

## Conclusion

A novel RDP driving principle was proposed to improve the performances of stick-slip actuators in terms of bidirectional motion consistency and linearity. Based on this principle, a novel RDP-type linear stick-slip actuator was developed to produce the pure rolling motion using the ITFM to generate the symmetric circular rotation. The symmetrical driving structure and the tangential contact condition enhance bidirectional consistency and reduce contact force fluctuation to ensure smoothness in output motion. Experiments were performed to demonstrate that the prototyped actuator could achieve excellent bidirectional motion consistency and linearity under both low and high driving frequencies with a single PZT, and generate sufficient output speed and load capacity, with no need for manual pre-adjustment processes. Based on this actuator, an MRI-compatible microsurgical instrument was developed, which successfully achieves opening and closing motions through single PZT actuation. The comprehensive cutting tests conducted on various specimens validate the instrument’s effectiveness and demonstrate its great potential for intraoperative MRI surgical applications. Furthermore, the presented novel rolling driving approach and RDP-type actuator provide an effective method to address the issues of bidirectional motion consistency and motion linearity with a single PZT. Future work will focus on its dynamic modeling, fiber Bragg grating (FBG)-based force sensing [39,40], and closed-loop control to extend the stick-slip actuator’s applications.

## Data Availability

All data needed to evaluate the conclusions are presented in the paper. Additional data related to this paper may be requested from the authors.
